# Metabolic Syndrome Components and the Risk of Colorectal Cancer: A Population-Based Prospective Study in Chinese Men

**DOI:** 10.3389/fonc.2019.01047

**Published:** 2019-10-17

**Authors:** Xin Li, Hongda Chen, Gang Wang, Xiaoshuang Feng, Zhangyan Lyu, Luopei Wei, Yan Wen, Shuohua Chen, Shouling Wu, Dong Hang, Min Dai, Ni Li, Jie He

**Affiliations:** ^1^Office of Cancer Screening, National Cancer Center/National Clinical Research Center for Cancer/Cancer Hospital, Chinese Academy of Medical Sciences and Peking Union Medical College, Beijing, China; ^2^Department of Oncology, Kailuan General Hospital, Tangshan, China; ^3^Health Department of Kailuan (Group), Tangshan, China; ^4^Department of Epidemiology, School of Public Health, Nanjing Medical University, Nanjing, China; ^5^Department of Thoracic Surgery, National Cancer Center/National Clinical Research Center for Cancer/Cancer Hospital, Chinese Academy of Medical Sciences and Peking Union Medical College, Beijing, China

**Keywords:** colorectal cancer, metabolic syndrome, cohort studies, prospective study, Chinese men

## Abstract

**Background:** To investigate the association between metabolic syndrome (MetS) and the risk of colorectal cancer (CRC) in Chinese men, this study was performed based on data from a large prospective cohort study conducted in China named the Kailuan men cohort study.

**Methods :** A total of 104,333 eligible men who participated in biennial examinations at least once from 2006 to 2015 were recruited. Cox proportional hazards regression models were used to estimate the effects of MetS components on CRC risk.

**Results:** During an 824,211.96 person-years follow-up, 394 CRC cases were verified. Participants with high waist circumference (≥90 vs. <90 cm) had a significantly higher risk of developing incident CRC (HR = 1.32, 95% CI: 1.07–1.64). Compared with participants with no MetS components, the HRs (95% CI) of developing CRC for men with 1, 2, and ≥3 MetS components were 1.53 (1.01–2.32), 1.42 (0.94–2.14), and 1.70 (1.12–2.56), respectively. In addition, a statistically significant trend (*P* for trend =0.04) of increased CRC risk with an increasing number of abnormal MetS components was observed. Furthermore, compared with no MetS components, the combination of high waist circumference and elevated fasting blood glucose along with normal levels of the other 3 components, showed a 126% increased risk of CRC.

**Conclusions:** Our study suggests that CRC risk is correlated with the number of abnormal MetS components in Chinese men. Men with high waist circumference and elevated fasting blood glucose may have a higher CRC risk even if they do not meet the MetS diagnostic criteria.

## Introduction

Colorectal cancer (CRC) is the fourth most common incident cancer around the world; 10.2% of the estimated 1,849,518 new CRC cases and 9.2% of the estimated 880,792 CRC-related deaths worldwide occurred in 2018 (1). In China, an estimated 376,300 new cases and 191,000 deaths from CRC occurred in 2015. Moreover, in recent decades, an increasing trend in the age-standardized incidence and mortality rates of CRC was observed among mainland Chinese men (2).

Metabolic syndrome (MetS) has been used to describe a composite condition characterized by a cluster of components, including high waist circumference (WC), elevated fasting blood glucose (FBG), elevated blood pressure (BP), elevated triglyceride (TG), and reduced high-density lipoprotein cholesterol (HDL-C). The increasing prevalence of MetS is now both a major clinical problem and a common public health problem (3). Several studies have suggested that MetS was related to an increased risk of cardiovascular disease (4, 5), type 2 diabetes mellitus (T2DM) (6), and all-cause mortality (7). Moreover, epidemiologic evidence has also suggested that the MetS may play a significant role in cancer development (8, 9).

Since 2001, several studies have investigated the association between MetS and CRC risk but have shown inconsistent results (10–12), especially among the Asian population (13). Additionally, evidence based on large prospective population studies is lacking in China. Moreover, it is not clear whether the risk of CRC could differ based on different combinations of MetS components, which is crucial for the risk stratification of individuals regarding the development of CRC.

Thus, we performed the current study with a large prospective cohort of Chinese men with the aim of examining the associations of MetS and its components with the risk of CRC. Mostly, we focused on both single and combinations of MetS components, which may be helpful in developing feasible prevention strategies for CRC.

## Methods

### Study Design and Population

The data were derived from a prospective dynamic cohort study of the Kailuan Group, which is a functional community in Tangshan City, Hebei Province in the northern region of China. Tangshan is located ~90 miles southeast of Beijing and is representative of the total population of China from a socioeconomic perspective (14). The Kailuan Group is a comprehensive company that manages machine manufacturing, the coal industry, coking, chemical production, transportation, healthcare, education, etc. Since May 2006, over 130,000 individuals were included in the biennial questionnaire interview and clinical examination at eleven affiliated hospitals in the Kailuan Group (15). The rationale and methodology of the Kailuan study have been described previously (16).

Participants who met the following criteria were recruited for the study: (1) men aged ≥18 years, (2) participants who provided informed consent and (3) individuals who completed the questionnaire interview. Participants without baseline information on WC (*n* = 3,786), FBG (*n* = 345), history of T2DM or treatment for T2DM (*n* = 427), BP (*n* = 135), history of or treatment for hypertension (*n* = 1,558), TG (*n* = 18), and HDL-C (*n* = 10) were excluded. Finally, a total of 104,333 participants were enrolled in the present study.

All participants provided written informed consent according to the guidelines of the Declaration of Helsinki, and the study was approved by the ethics committee of Kailuan General Hospital.

### Assessment of Exposure

In each biennial survey cycle, face-to-face questionnaire interviews and health examinations were facilitated by well-trained physicians or nurses using a standardized protocol to gather information on demographics, socioeconomic characteristics, lifestyle, medical history, and laboratory tests at baseline. Smoking was defined as at least one cigarette weekly for over 6 months, and participants were classified as “never smokers,” “former smokers,” or “current smokers.” The definition of alcohol drinking was the consumption of alcohol at least once per month for over 6 months, and participants were categorized as “never drinkers,” “former drinkers,” “ <1 time per day,” or “≥1 time per day.” Sitting time was categorized as “ <4 h per day, “4–8 h per day,” and “≥8 h per day.” WC was measured horizontally at the midpoint between the anterior superior iliac crest and the inferior rib. Systolic BP and diastolic BP levels were taken on the left arm using a mercury sphygmomanometer with a cuff of suitable size after participants rested for 5 min, which followed the standard recommended procedures. Two readings were taken at a 5-min interval, and the mean value of the two measurements was recorded for data analysis (17).

Morning fasting blood samples from each participant were examined using a standard operating procedure, and FBG levels were determined by the hexokinase/glucose-6-phosphate dehydrogenase method. The coefficient of variance of blind quality control samples was < 2.0%. TG levels were detected by the glycerol phosphate oxidase (GPO) method (interassay coefficient of variance <10%), and HDL-C levels were detected in the supernatant after the sediment of apolipoprotein B-containing lipoproteins with dextran sulfate and magnesium chloride (15). All blood samples were measured using an autoanalyzer (Hitachi 747; Hitachi, Tokyo, Japan) (18).

### Definition of Metabolic Syndrome

We defined MetS according to the American Heart Association, and National Heart, Lung, and Blood Institute (AHA/NHLBI) criteria in 2009 (≥ 3 of the necessary 5 criteria): (a) high WC levels (≥90 cm), (b) elevated FBG levels (or antidiabetic medication for the treatment of previously elevated glucose) (≥5.6 mmol/L), (c) elevated BP levels (or antihypertensive medication treatment for previous hypertension) (systolic≥130 or diastolic≥85 mm Hg), (d) elevated TG levels (≥1.7 mmol/L) and (e) reduced HDL-C levels (<1.03 mmol/L) (3).

### Assessment of Outcomes

The endpoint was newly diagnosed cancer, death, or administrative censoring (December 31, 2015), whichever came first. For the duration of the follow-up, new cases were identified by tracking participants once they participated in regular questionnaires and health check-ups biennially. In addition, incident cancer cases were assessed annually by examining medical records linked with the Tangshan medical insurance system, and discharge summaries were also tracked from the eleven affiliated hospitals. Furthermore, death certificates from the Kailuan social security system were obtained to further confirm the outcome information (19).

The CRC cases were determined by study physicians' review of medical records. Information on pathological diagnoses, imaging diagnoses (including magnetic resonance imaging and computerized tomographic scanning), and blood biochemical examination were collected to assess incident CRC cases. Cancer was coded according to the International Classification of Diseases, tenth revision (ICD-10), and CRC was coded as C18-C20.

### Statistical Analysis

Proportions and Chi-square tests were used to describe and compare categorical variables. The mean (standard deviation, SD) and *T*-test were used to describe and compare continuous variables. Person-years of risk was calculated from the date of recruitment until the date of first incident cancer, death, or end of follow-up (December 31, 2015), whichever occurred first. We used multivariable Cox proportional hazards regression models to estimate the association between MetS and MetS components with the risk of developing CRC. Model 1 was adjusted for age (continuous). Model 2 was additionally adjusted for income (<500 Chinese Yuan (CNY) per month, 500–1,000 CNY per month, or ≥1,000 CNY per month), education level (illiterate/primary school, junior high school, senior high school, or college and above), frequency of tobacco smoking (never smokers, former smokers, or current smokers), frequency of alcohol drinking (never drinkers, former drinkers, <1 time per day, or ≥1 time per day), and sitting time (<4 h per day, 4–8 h per day, or >8 h per day). Tests for linear trends across quartiles of MetS were performed by modeling the categories as continuous variables. To evaluate the potential effect of various combinations of MetS components, we conducted multivariable-adjusted risk of CRC development associated with combinations of MetS components, compared to participants with no components. Sensitivity analyses were performed to evaluate the consistency of our findings. First, we repeated the main analyses after excluding participants with a history of taking antidiabetic medication or a history of antihypertensive medication use; second, to examine the possibility of reverse causation, we excluded CRC cases that occurred during the first 3 years of follow-up.

All data management and statistical analyses were conducted using SAS, version 9.4 (SAS Institute Inc, Cary, NC, USA). The statistical tests were two-sided, and a *P* value < 0.05 was considered statistically significant.

## Results

### Baseline Characteristics of the Baseline Study Participants

By December 2015, a total of 104,333 eligible men participants with a mean age of 51.2 years were recruited for this study. During a median follow-up of 8.9 years, 394 incident CRC cases were verified over a total of 824,211.96 person-years. The mean values of WC, FBG, DBP, SBP, TG, and HDL-C were 87.97 cm, 5.54 mmol/L, 84.64 mmHg, 132.21 mmHg, 1.72 mmol/L, and 1.51 mmol/L, respectively. As shown in Table 1, compared to participants without CRC, incident CRC cases were more likely to be older, have lower education levels (illiterate or primary school) and were more prone to alcohol drinking (all *P* values < 0.05). The levels of WC (*P* < 0.001), FBG (*P* = 0.05), and SBP (*P* < 0.001) were higher among participants with CRC than among those without CRC. However, the levels of DBP (*P* = 0.68), TG (*P* = 0.99), and HDL-C (*P* = 0.16) were similar between the two groups (Table 1).

**Table 1:** Baseline characteristics of men by CRC, Kailuan cohort, 2006–2015.

**Characteristics**	**Total cohort**	**CRC**		***P* value**
	**(*n* = 104,333)**	**Yes (*n* = 394)**	**No (*n* = 103,939)**	
WC (cm)^a^	87.97 (9.68)	89.94 (9.86)	87.96 (9.68)	<0.001
Age (years)^a^	51.21 (13.46)	59.84 (9.89)	51.17 (13.46)	<0.001
FBG (mmol/L)^a^	5.54 (1.70)	5.74 (2.00)	5.54 (1.70)	0.05
DBP (mmHg)^a^	84.64 (11.72)	84.89 (11.40)	84.66 (11.73)	0.68
SBP (mmHg)^a^	132.21 (20.66)	136.02 (21.41)	132.20 (20.65)	<0.001
TG (mmol/L)^a^	1.72 (1.53)	1.72 (1.22)	1.72 (1.53)	0.99
HDL-C (mmol/L)^a^	1.51 (0.45)	1.54 (0.44)	1.51 (0.45)	0.16
Education level^b^				
Illiterate or primary school	11,978 (11.46)	75 (19.04)	11,903 (11.26)	<0.0001
Junior high school	67,605 (66.75)	265 (67.26)	69,305 (66.74)	
Senior high school	14,524 (13.93)	38 (9.64)	14,486 (13.95)	
College and above	8,158 (7.83)	16 (4.06)	8,142 (7.84)	
Income (yuan per month)^b^				
< 500	25,186 (25.12)	88 (23.10)	25,098 (25.13)	0.006
500–1,000	55,668 (55.52)	240 (62.99)	55,428 (55.49)	
≥ 1,000	19,409 (19.36)	53 (13.91)	19,356 (19.38)	
Smoking status				
Never	54,435 (56.43)	199 (56.06)	54,236 (56.43)	0.35
Former	3,986 (4.13)	20 (5.63)	3,966 (4.13)	
Current	38,052 (39.44)	136 (38.31)	37,916 (39.45)	
Alcohol drinking status^b^				
Never	52,228 (50.07)	192 (48.73)	52,036 (50.08)	<0.0001
Former	4,462 (4.28)	18 (4.57)	4,444 (4.28)	
<1 time/day	25,790 (24.73)	65 (16.50)	25,273 (24.76)	
≥1 time/day	21,823 (20.92)	119 (30.20)	21,704 (20.89)	
Sitting time (h/day)^b^				
<4	72,543 (72.27)	293 (74.74)	72,250 (72.26)	0.07
4–8	24,526 (24.43)	94 (23.98)	24,432 (24.44)	
≥8	3,310 (3.30)	5 (1.28)	3,305 (3.31)	

*CRC, colorectal cancer; WC, waist circumference; FBG, fasting blood glucose; BP, blood pressure; TG, triglyceride; HDL-C, high-density lipoprotein cholesterol*.

a *Mean (standard deviation), P values from T-test*.

b *N (%), P values from the χ^2^ test*.

### Associations Between Individual Components of MetS and CRC Risk

In multivariable-adjusted models, compared to the participants with normal WC (<90 cm), men with a high WC (≥90 cm) had a significantly higher risk of developing incident CRC (HR = 1.32, 95% CI: 1.07–1.64). Men with elevated FBG (≥5.6 mmol/l or antidiabetic medication for the treatment of previously elevated glucose vs. < 5.6 mmol/1) had an increased risk of CRC with borderline significance (HR = 1.19, 95% CI: 0.96–1.48). Men with elevated TG (≥ 1.7 mmol/1 vs. < 1.7 mmol/1) and reduced HDL-C (<1.03 mmol/L vs. ≥1.03 mmol/L) had an increased but not statistically significant risk of CRC development (HR = 1.12, 95% CI: 0.90–1.41; HR = 1.24, 95% CI: 0.84–1.84, respectively). No increased risk was observed for men with elevated BP (SBP ≥130 or DBP ≥85 mm Hg or antihypertensive medication treatment for previous hypertension vs. normal) (HR = 1.04, 95% CI: 0.82–1.31; Table 2).

**Table 2:** Associations between individual components of MetS and CRC risk in men, Kailuan cohort, 2006–2015.

**Groups**	**Cases**	**Person-years**	**HR (95% CI)**	
			**Model 1^**a**^**	**Model 2^**b**^**
**WC (cm)**				
<90	190	479,112.87	ref	ref
≥90	204	345,099.09	1.27 (1.05–1.55)	1.32 (1.07–1.64)
**FBG (mmol/l)**^**c**^				
Normal	242	562,176.91	ref	ref
High	152	262,035.04	1.26 (1.03–1.55)	1.19 (0.96–1.48)
**BP (mmHg)**^**d**^				
Normal	123	333,917.25	ref	ref
High	271	490,294.71	1.05 (0.85–1.31)	1.04 (0.82–1.31)
**TG (mmol/l)**				
<1.7	259	553,472.89	ref	ref
≥1.7	135	270,739.07	1.18 (0.96–1.46)	1.12 (0.90–1.41)
**HDL-C (mmol/l)**				
<1.03	33	59,545.17	1.26 (0.88–1.80)	1.24 (0.84–1.84)
≥1.03	361	764,666.79	ref	ref

*CRC, colorectal cancer; MetS, metabolic syndrome; WC, waist circumference; FBG, fasting blood glucose; BP, blood pressure; TG, triglyceride; HDL-C, high-density lipoprotein cholesterol*.

a *Adjusted for age*.

b *Adjusted for age, education level, income status, frequency of tobacco smoking, frequency of alcohol drinking, and sitting time*.

c *High was defined as FBG levels ≥5.6 mmol/L or anti-diabetic medication for the treatment of previously elevated glucose. Normal was defined as FBG <5.6 mmol/L*.

d *High was defined as systolic blood pressure ≥130 or diastolic blood pressure ≥85 mm Hg or antihypertensive medication treatment for previously hypertension, Normal was defined as systolic blood pressure < 130 and diastolic blood pressure <85 mm Hg*.

### Association Between MetS and CRC Risk

Multivariate analysis showed that compared to men with no MetS components, the HRs (95% CI) for developing CRC among men with 1, 2, and ≥3 MetS components were 1.53 (1.01–2.32), 1.42 (0.94–2.14), and 1.70 (1.12–2.56), respectively, which showed a statistically significant trend (*P* for trend = 0.04) of increased CRC risk with an increasing number of abnormal MetS components. In addition, when compared to those who did not meet the MetS diagnostic criteria (no. of MetS components <3), men with MetS had an increased risk of CRC with borderline significance (HR = 1.22, 95% CI: 0.97–1.53; Table 3).

**Table 3:** Associations between MetS and CRC risk in men, Kailuan cohort, 2006–2015.

**Groups**	**Cases**	**Person-years**	**HR (95% CI)**	
			**Model 1^**a**^**	**Model 2^**b**^**
**No. of MetS components**^**c**^				
0	33	131,781.60	ref	ref
1	112	237,461.25	1.48 (1.00–2.19)	1.53 (1.01–2.32)
2	118	240,331.66	1.41 (0.96–2.08)	1.42 (0.94–2.14)
≥3	131	214,637.45	1.74 (1.18–2.55)	1.70 (1.12–2.56)
*P* for trend			0.01	0.04
**Dichotomously defined**				
No MetS	263	609,574.51	ref	ref
MetS	131	214,637.45	1.27 (1.03–1.56)	1.22 (0.97–1.53)

*CRC, colorectal cancer; MetS, metabolic syndrome; No, number*.

a *Adjusted for age*.

b *Adjusted for age, education level, income status, frequency of cigarette smoking, frequency of alcohol drinking, and sitting time*.

c *Cut-off points were based on the American Heart Association, and National Heart, Lung, and Blood Institute (AHA/NHLBI) criteria in 2009 (≥ 3 of the necessary 5 criteria): (a) high WC levels (≥90 cm), (b) elevated FBG levels (or antidiabetic medication for the treatment of previously elevated glucose) (≥5.6 mmol/L), (c) elevated BP levels (or antihypertensive medication treatment for previous hypertension) (systolic ≥ 130 or diastolic ≥ 85 mm Hg), (d) elevated TG levels (≥1.7 mmol/L), (e) reduced HDL-C levels (<1.03 mmol/L). MetS was defined as the presence of ≥ 3 of the necessary 5 criteria*.

### Combined Associations of MetS Components With CRC Risk

We examined the HRs of all combinations (excluding 4 combinations owing to the limited number of cases) of the 5 MetS components. The no MetS component group was used as the reference category, and a combination of high WC and elevated FBG with normal levels of the other 3 components was associated with an increased risk for CRC (HR = 2.26, 95% CI: 1.10–4.65). In addition, the combination of high WC, elevated FBG and elevated BP (HR = 1.82, 95% CI: 1.08–3.08); the combination of high WC, elevated FBG and elevated TG (HR = 2.66, 95% CI: 1.21–5.81); and the combination of high WC, elevated BP and reduced HDL-C (HR = 2.75, 95% CI: 1.06–7.12) were associated with a higher risk of developing CRC (Table 4).

**Table 4:** Associations of MetS component combinations with CRC risk in men, Kailuan cohort, 2006–2015.

**Combination of components**			**Cases**	**Person-years**	**HR (95% CI)**	
					**Model 1^**a**^**	**Model 2^**b**^**
Null			33	131,781.60	ref	ref
WC↑	FBG↑		10	15,809.74	1.96 (0.97–4.01)	2.26 (1.10-4.65)
WC↑	BP↑		47	78,214.68	1.47 (0.93–2.31)	1.50 (0.93–2.43)
WC↑	TG↑		8	23,375.18	1.32 (0.61–2.87)	1.51 (0.69–3.32)
WC↑	HDL-C↓		1	4,477.33	0.82 (0.11–6.01)	0.97 (0.13–7.15)
FBG↑	BP↑		23	51,737.01	1.22 (0.72–2.09)	1.08 (0.60–1.95)
FBG↑	TG↑		5	12,748.92	1.57 (0.61–4.03)	1.42 (0.50–4.04)
FBG↑	HDL-C↓		0	–	–	–
BP↑	TG↑		20	42,342.43	1.56 (0.90–2.73)	1.45 (0.80–2.64)
BP↑	HDL-C↓		3	6,246.89	1.32 (0.40–4.32)	1.56 (0.47–5.13)
TG↑	HDL-C↓		1	3,171.50	1.59 (0.22–11.61)	1.88 (0.26–13.81)
WC↑	FBG↑	BP↑	33	43,628.16	1.85 (1.13–3.01)	1.82 (1.08–3.08)
WC↑	FBG↑	TG↑	9	12,425.13	2.63 (1.26–5.50)	2.66 (1.21–5.81)
WC↑	FBG↑	HDL-C↓	1	1,107.33	–	–
WC↑	BP↑	TG↑	22	53,784.33	1.20 (0.70–2.06)	1.13 (0.62–2.04)
WC↑	BP↑	HDL-C↓	5	6,054.71	2.21 (0.86–5.68)	2.75 (1.06–7.12)
WC↑	TG↑	HDL-C↓	1	3,238.83	1.33 (0.18–9.70)	1.61 (0.22–11.83)
FBG↑	BP↑	TG↑	12	26,834.86	1.43 (0.74–2.78)	1.32 (0.64–2.72)
FBG↑	BP↑	HDL-C↓	1	2,192.96	1.22 (0.17–8.93)	1.52 (0.21–11.18)
FBG↑	TG↑	HDL-C↓	0	–	–	–
BP↑	TG↑	HDL-C↓	0	–	–	–
All of 5			7	4625.48	4.41 (1.95–9.98)	4.55 (1.88–11.00)

*CRC, colorectal cancer; MetS, metabolic syndrome; WC, waist circumference; FBG, fasting blood glucose; BP, blood pressure; TG, triglyceride; HDL-C, high-density lipoprotein cholesterol*.

WC↑, high WC; FBG↑, elevated FBG levels; BP↑, elevated BP levels; TG↑, elevated TG levels; HDL-C↓, reduced HDL-C levels.

a *Adjusted for age*.

b *Adjusted for age, education level, income status, frequency of cigarette smoking, frequency of alcohol drinking, and sitting time*.

### Sensitivity Analysis

In the sensitivity analysis, when excluding individuals with antidiabetic medication use history (*n* = 2,578, case no. = 19) and individuals with antihypertensive medication use history (*n* = 10,770, case no. = 59), the men with ≥3 MetS components (HR = 1.64, 95%CI:1.08–2.49, HR = 1.64, 95% CI: 1.07–2.51) still had a significantly increased risk of CRC development. After excluding CRC cases (no. = 99) that occurred during the first 3 years of follow-up, the results were similar (HR_3 components vs. 0 components_ = 1.54, 95% CI: 0.96–2.49; Table 5). The results of the sensitivity analyses did not alter the main findings.

**Table 5:** Sensitivity analysis of the association between MetS and CRC risk in men, Kailuan cohort, 2006–2015.

**No. of MetS components**	**Cases**	**Person-years**	**HR (95% CI)**	
			**Model 1^**a**^**	**Model 2^**b**^**
**Excluding men with antidiabetic medication use history**				
0	33	131,639.02	ref	ref
1	108	235,750.65	1.44 (0.97–2.13)	1.52 (1.00–2.29)
2	115	235,371.00	1.41 (0.96–2.08)	1.44 (0.95–2.17)
≥3	119	202,361.22	1.70 (1.16–2.51)	1.64 (1.08–2.49)
*P* for trend			0.01	0.06
**Excluding men with antihypertensive medication use history**				
0	33	131,781.60	ref	ref
1	107	223,255.16	1.55 (1.05–2.29)	1.59 (1.05–2.42)
2	97	212,505.33	1.36 (0.92–2.02)	1.35 (0.89–2.07)
≥3	98	172,899.43	1.69 (1.14–2.51)	1.64 (1.07–2.51)
*P* for trend			0.05	0.12
**Excluding men who developed CRC within the first 3 years of follow-up**				
0	25	131,590.14	ref	ref
1	86	236,940.80	1.52 (0.97–2.37)	1.57 (0.97–2.52)
2	92	239,801.29	1.47 (0.94–2.29)	1.48 (0.92–2.37)
≥3	92	214,219.48	1.64 (1.05–2.55)	1.54 (0.96–2.49)
*P* for trend			0.08	0.24

*CRC, colorectal cancer; MetS, metabolic syndrome; No, number*.

a *Adjusted for age*.

b *Adjusted for age, education level, income status, frequency of cigarette smoking, frequency of alcohol drinking, and sitting time*.

## Discussion

Based on this large prospective cohort study among Chinese men, we documented that high WC was associated with CRC development. When considered combinedly, the number of abnormal MetS components was linearly associated with increased CRC risk. Notably, among MetS components, the combination of high WC and elevated FBG may be associated with an increased CRC risk, even in individuals who did not meet the MetS diagnostic criteria. To our knowledge, this is the first large prospective cohort study to report the associations of both single and combinations of components of MetS with CRC risk among men in China, and the results could provide powerful evidence supporting a potential effect of MetS components on increased CRC risk.

The evidence of the associations between individual components of MetS and CRC risk differed as follows. The increased risk of CRC in men with high WC in this study was similar to that observed in previous studies (10, 20, 21). The prospective cohort study Atherosclerosis Risk in Communities (ARIC) in the United States showed that compared with men with a WC <102 cm, those with abdominal obesity (WC >102 cm) had a higher risk of CRC (RR = 1.40; 95% CI, 1.00–1.90) (10). A meta-analysis of seven prospective cohort studies in Europe revealed that WC was associated with the development of CRC among men (per 1-s.d. increment: HR = 1.21, 95% CI:1.08–1.35) (20).

The Korean Metabolic Syndrome Research Initiative study involving 175,677 participants with a mean follow-up of 4.7 years found that elevated FBG was associated with higher CRC risk in men (HR = 1.51, 95% CI:1.12–2.05) (22). However, the Metabolic Syndrome and Cancer Project (Me-Can) of 289,866 adult men in Europe with a mean follow-up of 12.8 years showed no statistically significant association between CRC risk and FBG level in men (23). In most previous studies, hypertension has been related to a non-significant increased risk of CRC incidence (11, 24). Consistent with a cohort study in Austria (25) and the Alpha-Tocopherol, Beta-Carotene Cancer Prevention (ATBC) Study cohort (26), we found no significant association between CRC and elevated TG or reduced HDL-C.

Our observation of the association of CRC risk with MetS is consistent with the results of previous studies (10–12, 27–30). A meta-analysis of 15 datasets of men found a significantly positive association between MetS and CRC (RR = 1.33, 95% CI: 1.18–1.50) as compared with those without MetS (13). Moreover, in 2006, the ARIC multicenter prospective cohort study involving 14,109 men reported a relationship of MetS based on high WC, elevated TG, reduced HDL-C, elevated BP and elevated FBG with CRC risk (3 vs. 0 components, RR = 1.78, 95% CI: 1.0–3.6; ≥3 components vs. <3 components, RR = 1.31,95% CI: 0.9–1.9) (10). The Physicians' Health Study also found that among men, each additional metabolic abnormality increased the risk of CRC (RR = 1.16, 95% CI: 1.05–1.29) (11). Taken together, the results of the present study could be reasonable.

Several potential mechanisms might explain the associations between MetS components and the development of CRC (31). First, it has been proposed that MetS is represented by insulin resistance and hyperinsulinemia (32), and insulin resistance could stimulate the production of reactive oxygen species (ROS), which may damage DNA and contribute to malignant transformation (33). In addition, hyperinsulinemia leads to decreased hepatic synthesis of insulin-like growth factor (IGF)-binding protein 1 (IGFBP-1) and protein 2 (IGFBP-2) and may result in increased bioactivity of IGF-I (34, 35), which directly induces the development of CRC due to its mitogenic and antiapoptotic actions (33). Moreover, obese people have downregulated expression of adiponectin, an adipokine with anti-inflammatory and insulin-sensitizing properties, which can play an etiologic role in mutagenesis and may contribute to the development of cancer (33, 36). However, some evidence has identified obesity-related inflammation as a factor involved in cancer development, but more research is needed to confirm the extent of the association of factors that may be mediated by MetS (37, 38). Hence, more research is needed to better elucidate the potential biological mechanism underlying the relationship between MetS components and CRC.

In our study, the combined effects of the 5 MetS components on the risk of CRC development varied; in particular, the coexistence of high WC and elevated FBG exhibited a significant association with higher CRC risk. The different associations of the combinations of the MetS components with CRC risk highlight the role of the combination of high WC and elevated FBG. This finding might have key clinical and scientific significance, as it indicates that individuals who have two components of MetS, such as high WC and elevated FBG, should receive attention, even when they do not meet the MetS diagnostic criteria. In addition, the combination of high WC, elevated BP and reduced HDL-C was associated with a significantly higher risk of developing CRC; however, the result may be unstable because of the small number of cases.

Our study has several strengths. One of the main strengths is its prospective design and the large sample size, which resulted in sufficient statistical power to detect modest differences and minimized the underlying bias due to preclinical disease. In addition, the laboratory tests (e.g., TG, HDL-C, and FBG) were conducted on the basis of standard procedures, which minimized the detection bias. Moreover, the WC and BP measurements were obtained by trained physicians or nurses rather than self-report, avoiding misclassification bias. However, there are some limitations that should be considered when interpreting the results. The relatively short follow-up time (median, 8.9 years) is the major limitation of our study and precluded subtype analysis of CRC because of a limited number of cases. Additionally, in some groups, the small number of cases may have led to unstable results, and due to the limited number of cases, the four combinations of MetS components were not assessed. However, the Kailuan cohort is an ongoing dynamic cohort study; further follow-up should be conducted to identify more cases, and more stable results can be expected.

In conclusion, our study suggests that CRC risk is correlated with the number of abnormal MetS components in Chinese men. In addition, the combination of high WC and elevated FBG may have both clinical and public health significance for identifying individuals with high CRC risk. Therefore, the control of MetS, especially maintaining WC and FBG within an appropriate range, may be a meaningful primary prevention strategy to decrease CRC risk among Chinese men.

## Data Availability Statement

The datasets for this manuscript are not publicly available because all our data are under regulation of both the National Cancer Center of China and Kailuan Group. Requests to access the datasets should be directed to JH, hejie@cicams.ac.cn and SW, drwusl@163.com.

## Ethics Statement

The studies involving human participants were reviewed and approved by The ethic committee of the Kailuan General Hospital. The patients/participants provided their written informed consent to participate in this study.

## Author Contributions

XL, NL, GW, SW, MD, and JH: conception and design. NL, MD, and JH: development of methodology and study supervision. XL, HC, XF, SC, LW, ZL, and YW: acquisition of data. XL, HC, and DH: analysis and interpretation of data. XL, NL, MD, and JH: drafting, review, and/or revision of the study. All authors approved the final version of the study.

### Conflict of Interest

The authors declare that the research was conducted in the absence of any commercial or financial relationships that could be construed as a potential conflict of interest.

## Abbreviations

CRC: colorectal cancer
MetS: metabolic syndrome
WC: waist circumference
FBG: fasting blood glucose
BP: blood pressure
TG: triglyceride
HDL-C: high-density lipoprotein cholesterol
T2DM: type 2 diabetes mellitus
GPO: glycerol phosphate oxidase
AHA/NHLBI: American Heart Association, and National Heart, Lung, and Blood Institute
ICD-10: International Classification of Diseases, tenth revision
CI: confidence interval
CNY: Chinese Yuan
ARIC: Atherosclerosis Risk in Communities
Me-Can: the metabolic syndrome and cancer project
ROS: reactive oxygen species
IGF: insulin-like growth factor
IGFBP: insulin-like growth factor-binding proteins
ATBC: Alpha-Tocopherol, Beta-Carotene Cancer Prevention.
